# Size and Charge Evaluation of Standard Humic and Fulvic Acids as Crucial Factors to Determine Their Environmental Behavior and Impact

**DOI:** 10.3389/fchem.2018.00235

**Published:** 2018-07-05

**Authors:** Martina Klučáková

**Affiliations:** Faculty of Chemistry, Materials Research Centre, Brno University of Technology, Brno, Czechia

**Keywords:** humic acids, fulvic acids, solubility, stability, particle size, charge

## Abstract

In this work, the size and charge of humic and fulvic standards purchased from the International Humic Substances Society are presented and discussed. The secondary structure of humic substances in water environment as well as the size and shape of the dissolved humic species and their changes are ill-defined, very dynamic and can be strongly affected by environmental conditions as the concentration, pH, and ionic strength. They have a strong propensity to aggregate which control their interactions with other components, mobility, and functioning in the environment. Particle size distributions were determined by means of dynamic light scattering, zeta potential by Doppler electrophoresis. The intensity, volume, and number particle size distribution were obtained. Two or three different size fractions were detected in the studied systems. Large macroparticles (>1 μm) were observed in the majority of them, mainly in the case of more concentrated solutions. Medium fractions of fulvic submicroparticles had higher average diameters (500–1,200 nm) than those in humic acids (300–600 nm). Small nanoparticles (<100 nm) were detected mainly in alkaline solutions. Fulvic acids with more functional groups (active sites) can form more easily bigger particles mainly in medium concentration region. Alkaline conditions supported the expansion of humic and fulvic coils and liberation of small particles from them. The colloidal stability, indicated by more negative zeta potentials, was higher for humic acids. In the case of fulvic acids, the colloidal stability increased with increasing pH as a result of the dissociation of their functional groups. The increase of particle size corresponded usually with higher stability.

## Introduction

Humic and fulvic acids are the principal components of soil organic matter, peat, coal, sediments, and dissolved organic matter. They play indispensable roles in the environment in general (Tan, 2014; Klučákov and Kalina, 2015). The complex structure of humic and fulvic acids exhibiting a great diversity of functional groups makes an exact understanding of the mechanism of humic and fulvic interactions almost impossible. Their chemical and physicochemical behavior in natural soil and water environments is a function of their molecular structure, and dictates their organic matter mobility, interactions with clay surfaces, and aggregation in natural environments (Baalousha et al., 2006; Colombo et al., 2015; Klučáková and Věžníková, 2016).

The structure of humic and fulvic acids is dynamic with regard to environmental conditions. The concentration, pH, ionic strength, and nature of the counter-ion influence the size and shape of the dissolved organic species (Durce et al., 2016). However, many fundamental questions relating, in particular, to the physicochemical characteristics of humic and fulvic molecules are yet to be answered (Klučáková, 2016; Tarasevich et al., 2016). The data on the molecular mass and size of humic molecules reported in literature are rather confusing and are strongly influenced by differences in experimental conditions. Different methods do not give the same results. Differences in the obtained values have been attributed to either the variability of humic substances or the intrinsic limitations of methods when applied to poly-disperse humic systems (Piccolo, 2001; Klučáková and Věžníková, 2017).

Pinheiro et al. (1996) studied the dynamic properties of humic matter by dynamic light scattering and voltammetry. They stated that aggregates with different sizes co-existed for all studied samples and that their relative amounts, structure, and configuration depended on the sample preparation and its origin. Palmer and von Wandruszka (2001) showed that the evolution of particle sizes under different solution conditions progressed from “stretched out” anionic polymers at high pH, low cation concentrations, and low temperature, through micelle-like structures to colloidal precipitates as these conditions were changed. Manning et al. (2000) investigated the kinetics of the aggregation and precipitation of humic acids using multi-angle laser light scattering. They found that processes taking place over hours, days, or weeks need to be considered when reporting size and molecular mass. Chin et al. (1998) studied the spontaneous assembly of marine dissolved organic matter. They demonstrated the formation of polymer gels from free dissolved organic matter. Fujitake and Kawahigashi (1998) separated humic acids into six particle size fractions by gel permeation chromatography. Their results indicated that surface activity increased with increasing molecular weight or particle size. Manning et al. (2004) showed by means of laser diffraction that humic substances were capable of existing in dynamic equilibrium in which smaller (0.1–0.5 μm) and larger (3–1,000 μm) aggregates existed in a cycle of aggregation, precipitation, and re-solubilization. Experimental conditions influenced the size of the aggregates but did not deter the aggregation process. Sutton and Sposito (2005) published a new view on the molecular structure of humic substances. According to this, humic substances are collections of diverse, relatively low molecular mass components forming dynamic associations, stabilized by hydrophobic interactions and hydrogen bonds, and capable of organizing into a micellar structure in suitable aqueous environments. Baalousha et al. (2006) studied the influence of concentration, salt addition and pH on the conformation and size of humic substances using photon correlation spectroscopy and transmission electron microscopy. Their work provided evidence of a supramolecular structure composed of basic units of about 10 nm. Aggregation increased the size of the supramolecular network of humic substances Baigorri et al. (2007a) used ultrafiltration, size exclusion chromatography, and dynamic light scattering in order to analyze molecular size distribution changes induced by acidification. Their results indicated that humic substances appeared to be composed of two main fractions: a fraction which presented clear macromolecular behavior in solution, with macromolecules and/or very stable aggregates present; and another fraction that was principally formed by molecular aggregates (supramolecular associations), which also included molecules of low molecular weight and an unclear macromolecular nature. According to their finding, macromolecules, small molecules and supramolecular associations all seem to coexist in humic systems. In their other work (Baigorri et al., 2007b), humic substances were divided into three fractions. Gray humic substances had a clear macromolecular pattern. In fulvic acids, the coexistence of supramolecular assemblies and individual molecules was revealed. Brown humic acids contained both the macromolecular pattern and the supramolecular pattern. Jovanovic et al. (2013) confirmed that particle diameter and zeta potential were strongly influenced by humic concentration and pH value. Angelico et al. (2014) showed that particle size, charge, and colloidal stability were strictly dependent on surface functional groups. Tarasevich et al. (2016) divided humate particles into three fractions: nanoparticles, submicroparticles and microparticles. The characteristic diameter and zeta potential of the fractions were strongly dependent on concentration. Humate adsorbed on solid surfaces from dilute solutions contained particles less than 10 nm in size, whereas particles from 40 nm to several micrometers in size were detected for adsorption from more concentrated solutions. Similarly, Esfahani et al. (2015) categorized humic aggregates into three ranges: 10–100 nm, 100–1,000 nm, and >1 μm. Their zeta potential analysis demonstrated that colloidal stability increased as concentration increased.

The knowledge of particle size distribution and colloidal stability is very important for their functioning in nature. The mobility of any particle is strongly affected by its size. In the case of globular particles, their diffusion coefficient decreases with square of diameter. The aggregation of humic and fulvic particles can result in a loss of their mobility, potential sedimentation, and in the decrease of active sites accessible for pollutants in natural systems. The bioavailability of pollutants can be increased and decreased by the binding with humic and fulvic particles in the dependence on their size and shape. Some pollutants have ionic character, therefore the charges of humic and fulvic particles play an important role for their binding. In some cases, binding of humic substances can cause or support the aggregation. All these aspects can be taken in consideration in order to evaluate or predict the behavior of humic substances at given conditions.

In this complex work, the influence of the concentration of humic and fulvic standards on the size and charge of humic and fulvic acids was studied. In the case of fulvic acids, water and NaOH were used for the preparation of their solutions. Humic and fulvic acids extracted from different matrices were analyzed in this complex study.

## Materials and methods

### Humic and fulvic acids

Samples of humic and fulvic acids were purchased from the International Humic Substances Society Samples of Nordic lake humic acids (NLHA), Elliot soil humic acids (ESHA), Suwannee river humic acids (SRHA), Pahokee peat humic acids (PPHA), Leonardite humic acids (LEHA), Nordic lake fulvic acids (NLFA), Elliot soil fulvic acids (ESFA), Suwannee river fulvic acids (SRFA), and Pahokee peat fulvic acids (PPHA) were used in this study.

### Humic and fulvic solutions

Humic and fulvic acids were dissolved in a 0.01M solution of NaOH in order to achieve concentrations from 0.01 g.L^−1^ to 1 g.L^−1^. Fulvic acids were dissolved in water in the same concentration range. The solutions were stirred for 24 h. up to total dissolution. The obtained dissolve solutions were used for the determination of particle size distribution and zeta potential.

### Determination of particle size and charge

The particle sizes and zeta potentials of humic and fulvic acids samples were determined in their solutions by means of a Zetasizer Nano ZS with backscattering detection (Klučákov and Kalina, 2015; Klučáková and Věžníková, 2017). Dynamic light scattering technique measures the diffusion of particles moving under Brownian motion, and converts this to size and a size distribution using the Stokes-Einstein relationship. Laser Doppler Micro-electrophoresis is used for the measurement of zeta potential. An electric field is applied to a solution of molecules or a dispersion of particles, which then move with a velocity related to their zeta potential. This enables the calculation of electrophoretic mobility, and from this the zeta potential. All experiments were triplicated and average values are presented. The sizes of three populations presented in Table 1 have been determined on the basis of volume particle size distributions as their maxima. They are the average values of three measurements with standard errors.

**Table 1:** Average diameter of three volume based particle populations of humic and fulvic acids.

**Sample**	**Solvent**	**Concentration (g.L^−1^)**	**Population 1 (nm)**	**Population 2 (nm)**	**Population 3 (μm)**
NLHA	0.01M NaOH	0.01	91 ± 12	483 ± 34	
		0.1	66 ± 14	428 ± 98	
		1	37 ± 13	330 ± 58	4.8 ± 0.1
SRHA	0.01M NaOH	0.01	87 ± 7	560 ± 48	
		0.1	96 ± 27	507 ± 14	
		1	93 ± 20	559 ± 48	5.6 ± 0.1
PPHA	0.01M NaOH	0.01	73 ± 8	560 ± 40	4.8 ± 0.1
		0.1	48 ± 4	507 ± 34	5.3 ± 0.4
		1	26 ± 14	507 ± 6	5.0 ± 0.4
ESHA	0.01M NaOH	0.01	88 ± 11	563 ± 26	
		0.1	91 ± 18	512 ± 9	
		1	93 ± 22	563 ± 18	4.8 ± 0.3
LEHA	0.01M NaOH	0.01	63 ± 12	483 ± 42	5.6 ± 0.2
		0.1	55 ± 4	460 ± 12	5.6 ± 0.1
		1	63 ± 12	507 ± 80	5.6 ± 0.1
NLFA	0.01M NaOH	0.01	94 ± 11		
		0.1	79 ± 23	738 ± 15	5.6 ± 0.3
		1	58 ± 17	1164 ± 101	5.6 ± 0.2
SRFA	0.01M NaOH	0.01	80 ± 34	542 ± 21	
		0.1	73 ± 14	759 ± 55	5.6 ± 0.1
		1	88 ± 17	727 ± 58	5.6 ± 0.3
PPFA	0.01M NaOH	0.01	107 ± 15	1115 ± 79	5.6 ± 0.2
		0.1	83 ± 6	573 ± 42	5.6 ± 0.2
		1	58 ± 15	884 ± 68	5.3 ± 0.4
ESFA	0.01M NaOH	0.01	185 ± 46	1118 ± 43	5.6 ± 0.1
		0.1	78 ± 23	948 ± 35	5.6 ± 0.1
		1		1172 ± 153	5.6 ± 0.3
NLFA	water	0.01		1106 ± 145	5.2 ± 0.4
		0.1		1232 ± 178	5.6 ± 0.3
		1	63 ± 4	1006 ± 71	
SRFA	water	0.01		690 ± 21	5.6 ± 0.2
		0.1		559 ± 39	5.5 ± 0.3
		1	80 ± 12	690 ± 23	
PPFA	water	0.01		1327 ± 52	5.6 ± 0.3
		0.1	224 ± 18	890 ± 66	5.6 ± 0.1
		1	88 ± 15	1020 ± 12	5.6 ± 0.1
ESFA	water	0.01			3.1 ± 0.2
		0.1	106 ± 13	1085 ± 86	5.6 ± 0.1
		1	75 ± 23	620 ± 54	

## Results

**Figure 1:**
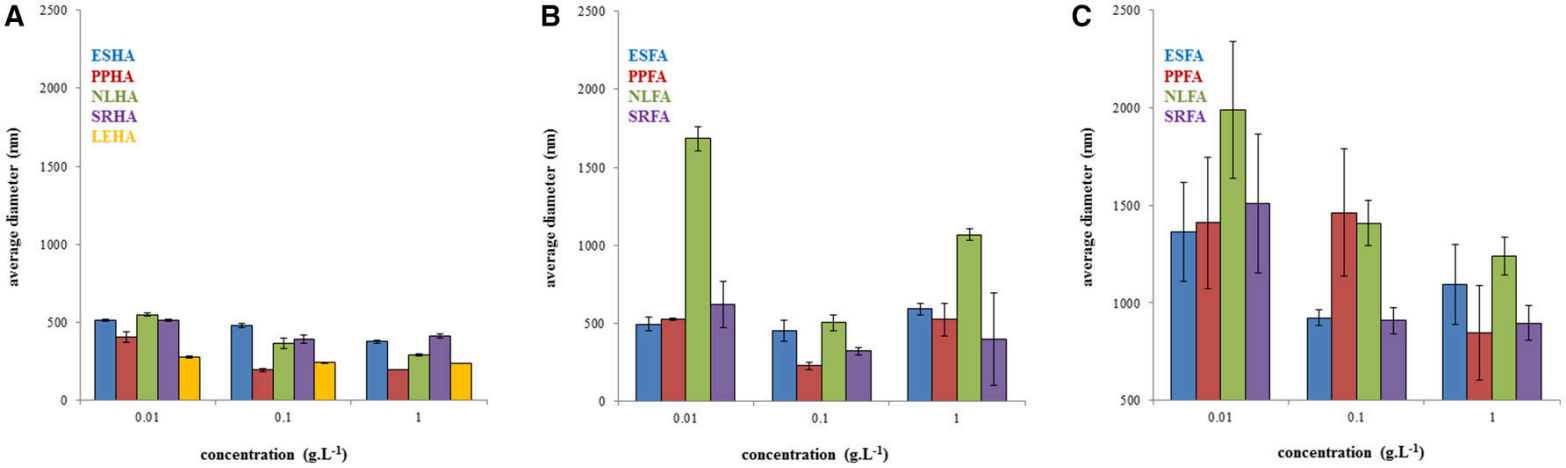
Average diameters of humic acids in 0.01M NaOH **(A)**, fulvic acids in 0.01M NaOH **(B)** and in water **(C)** determined on the basis of the intensity particle size distribution.

In Figure 1, the average size diameters for all studied solutions are shown. We can see that the determinations of average particle sizes were associated with very high errors. Average particle sizes were determined from intensity-based dynamic light scattering records and represent a single average, although the obtained records were multimodal.

**Figure 2:**
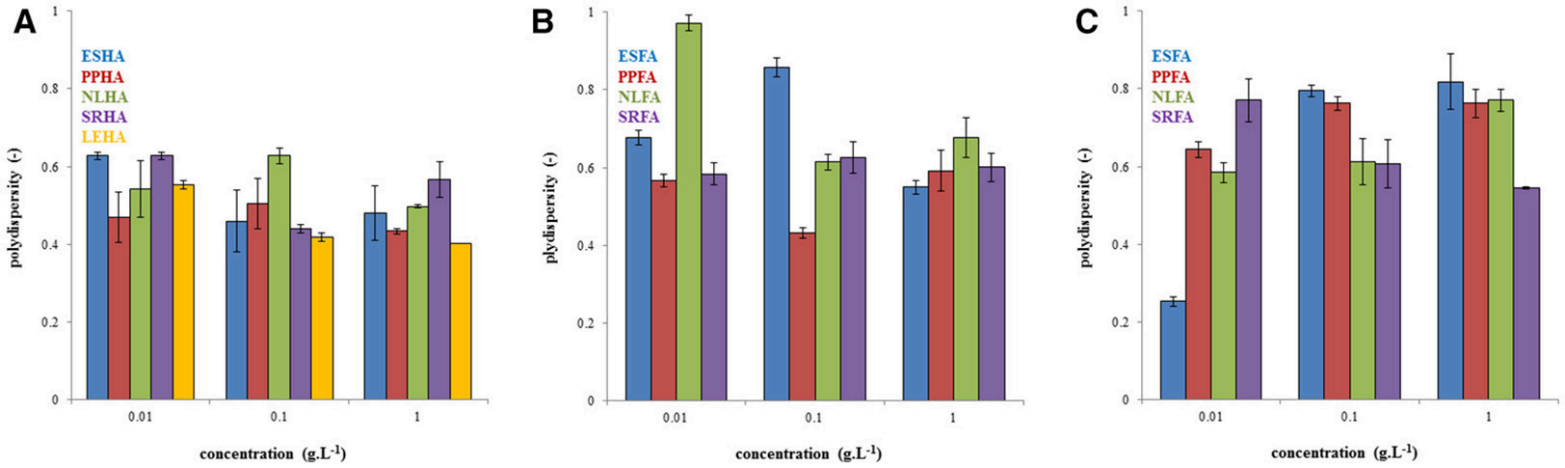
Polydispersity of humic acids dissolved in 0.01M NaOH **(A)**, fulvic acids dissolved in 0.01M NaOH **(B)**, and fulvic acids dissolved in water **(C)**.

The polydispersity of humic and fulvic acids is presented in Figure 2. In general, the polydispersity of humic acids was lower in comparison with fulvic acids. A maximum of polydispersity at concentration of 0.1 g.L^−1^ was observed for NLHA, PPHA, ESFA, and SRFA. No important trend in polydispersity was observed for humic and fulvic acids dissolved in NaOH. In the case of fulvic acids in water, the polydispersity increased with the increase of concentration with the exception of SRFA.

**Figure 3:**
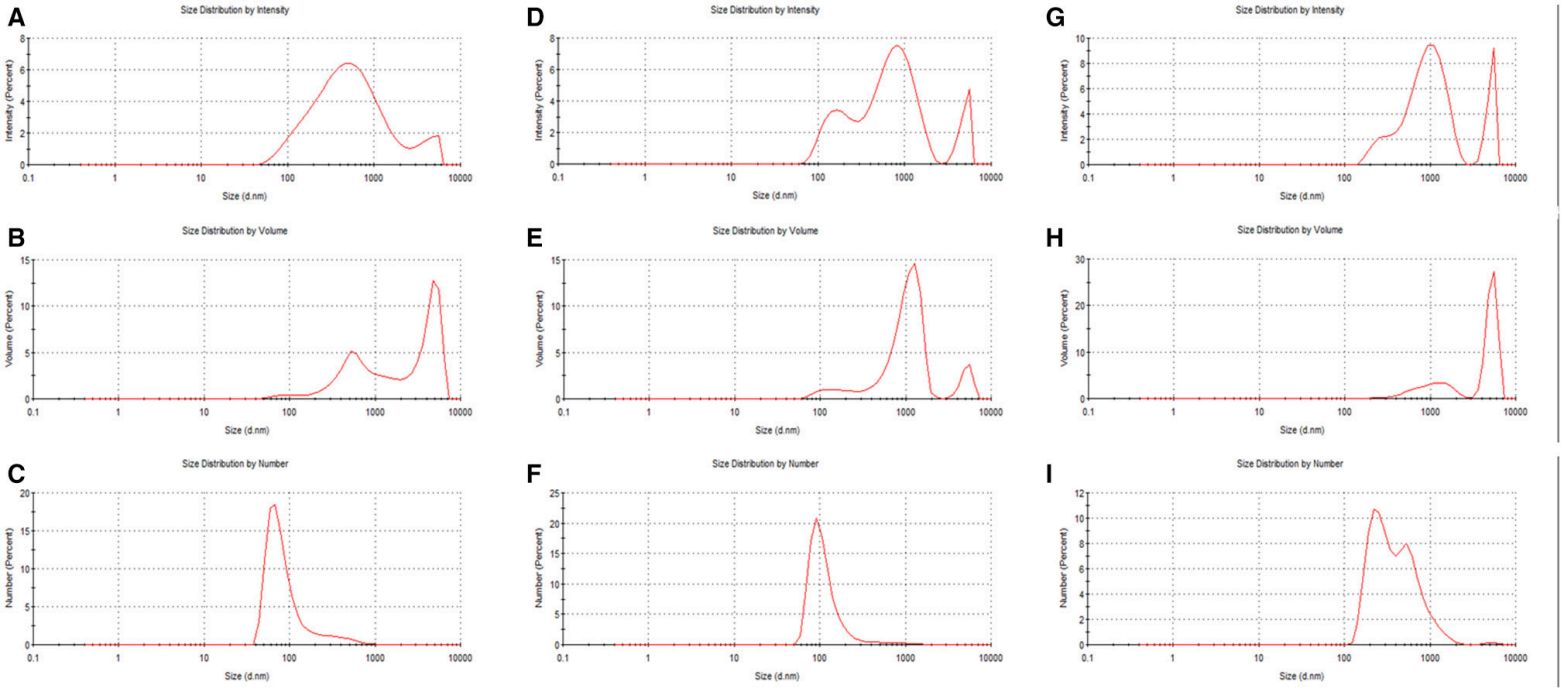
Intensity, volume and number particle size distributions of PPHA dissolved in 0.01M NaOH **(A–C)**, PPFA dissolved in 0.01M NaOH **(D–F)**, and PPFA dissolved in water **(G–I)**. The concentration of all solutions was 0.01 g dm^−3^.

In Figure 3, the example of the intensity, volume, and number particle size distributions of PPHA and PPFA is shown. While the intensity and volume size distributions of PPHA were bimodal, their number based distribution was mono-modal because the number of big particles occupying a large volume was very low. Similarly, the number-based mono-modal record obtained for PPFA in alkaline solution was also mono-modal, whereas the intensity and volume distributions had a tri-modal character. Records obtained for PPFA in water were different. A bimodal character was determined for all three distribution types. The sizes of the individual populations of PPFA in water were bigger in comparison with NaOH, and both types of PPFA solutions (in NaOH and water) contained bigger particles or aggregates than PPHA.

**Figure 4:**
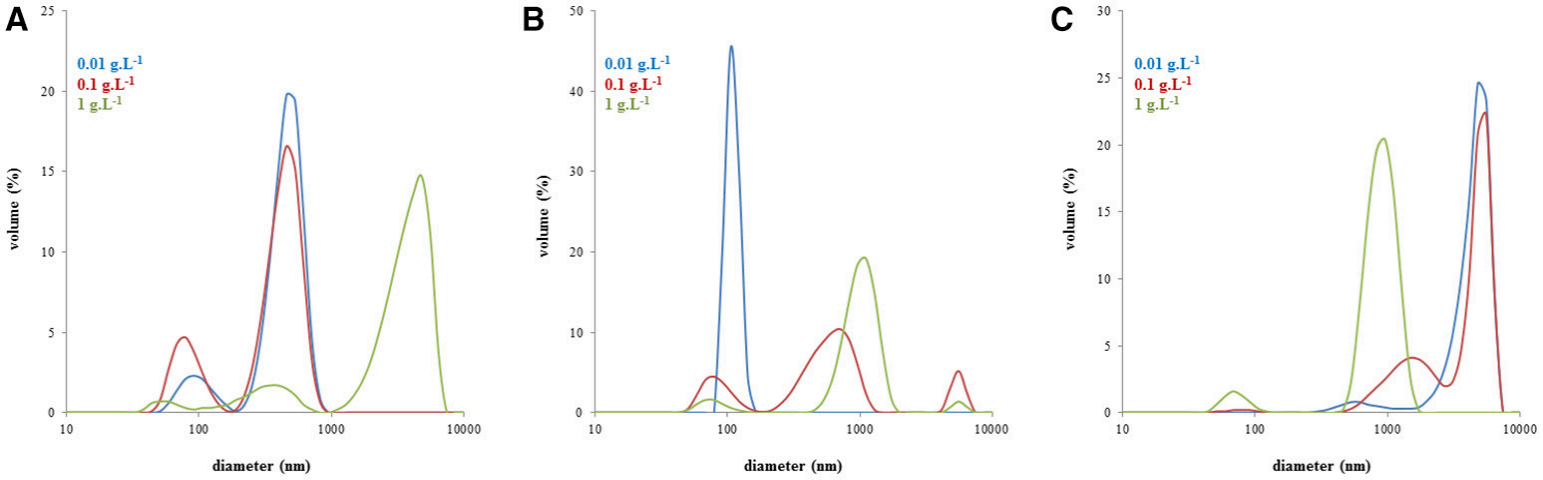
Volume particle size distributions of NLHA in dissolved 0.01M NaOH **(A)**, NLFA dissolved in 0.01M NaOH **(B)**, and NLFA dissolved in water **(C)**.

In Figure 4, changes in the volume based distributions of NLHA and NLFA with changing concentration are shown. NLHA samples dissolved in alkaline solution had a bimodal character at lower concentrations. Only the most concentrated NLHA solution contained a volume of big aggregates with diameter >1 μm. The record of the most concentrated NLHA solution had tri-modal character, but the volume occupied by smaller particles was much lower in comparison with more diluted solutions (here, medium fractions with a diameter of around 500 nm predominated). In contrast, the most diluted NLFA solution in NaOH had only one fraction with a diameter of around 100 nm and the volume occupied by bigger particles or aggregates was negligible. More concentrated alkaline solutions of NLFA had the main fraction with average diameters between 700 and 1,100 nm and included also significant volumes of small particles (70–80 nm) and big aggregates (5.5–5.6 μm). NLFA in water behaved differently. More diluted solutions contained mainly big aggregates (4.8–5.6 μm) and a lower volume of particles with diameters around 600 nm (0.01 g.L^−1^) and 1.5 μm (0.1 g.L^−1^). The most concentrated solution mainly consisted of particles with a diameter around 1 μm and contained a small volume of small particles (~70 nm).

The characteristic particle sizes of humic and fulvic acids are listed in Table 1. In general, the average diameter of the smallest particles of humic acids decreased with increasing concentration (NLHA, PPHA and LEHA) or remained at the same value (SRHA and ESHA). The size of the medium humic fraction also decreased (NLHA and PPHA) or fluctuated (SRHA, ESHA and LEHA). Macroparticles were observed mainly in more concentrated humic solutions (NLHA, SRHA and ESHA). In the case of PPHA and LEHA, microparticles were detected over the whole studied concentration range. Small nanoparticles were observed in all alkaline fulvic solutions except for the most concentrated ESFA solution. Their average diameters decreased (NLFA, PPFA, and ESFA) or fluctuated (SRFA) with increasing concentration. No submicroparticles or macroparticles were observed for diluted NLFA solutions. Macroparticles were detected in the majority of alkaline fulvic solutions. Small particles were observed only in more concentrated alkaline fulvic solutions as well as their water systems. Particles in the medium fraction had higher average diameters in comparison with fulvic acids dissolved in NaOH. The most concentrated solution of fulvic acids in water did not contain macroparticles except for PPFA. In contrast, the most diluted ESFA solution contained only macroparticles with a diameter around 3 μm.

**Figure 5:**
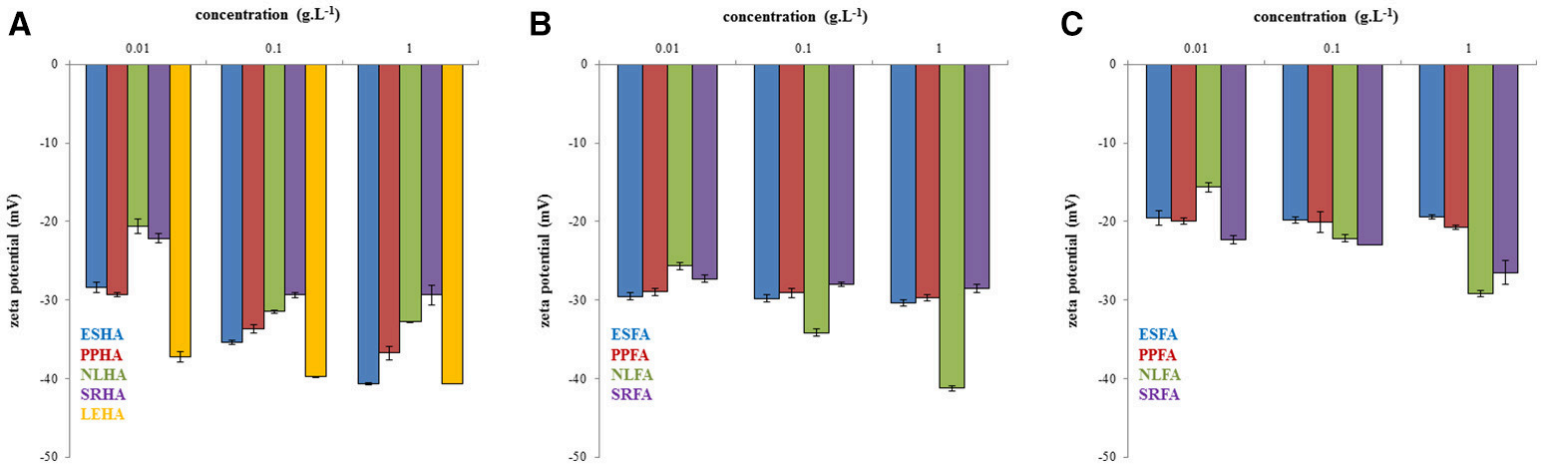
Zeta potential of humic acids dissolved in 0.01M NaOH **(A)**, fulvic acids dissolved in 0.01M NaOH **(B)** and fulvic acids dissolved in water **(C)**.

The zeta potentials of the studied systems are shown in Figure 5. More negative zeta potentials indicating higher stability and the presence of smaller humic particles or associates were determined in more concentrated humic solutions, while particle-size distribution was observed to have a bimodal character in diluted systems. As we can see, the zeta potential decreased generally with increasing concentrations of humic and fulvic acids. The lowest zeta potentials were determined for LEHA, ESHA, and NLFA in NaOH, the highest for ESFA and PPFA in water.

## Discussion

Our results presented in Figure 1 confirmed that average size diameter is not a suitable parameter for characterizing humic substances and should be used only conditionally as the apparent mean particle size in comparisons with other works. This conclusion was supported by the determination of polydispersity (see Figure 2), which was very high. There results were caused by multi-modal character of dynamic light scattering records which contained two or three populations of humic substances. It means that humic substances contained two or three size fractions with different mobility. Therefore, interactions of these different fractions can affect the bioavailability of pollutants by different way. Their binding with small dissolved particles can support the mobility of pollutants in some cases and their bioavailability depends of the bond strength between pollutant and humic substance. The complexation of pollutants by bigger humic particles is usually connected with lower mobility. It is necessary to take account of the possibility of aggregation of humic particles and their sedimentation as a result of their interactions with pollutants. If we compare the mean particle sizes of humic and fulvic acids, we can see that humic particles seem much smaller that fulvic ones. This is surprising because the traditional approach to the fractionation of humic substances defines humic acids as bigger than fulvic ones. One reason could be the easier aggregation of fulvic acids, which is seemingly more intensive in water. A further interesting result is the decrease in mean particle size with increasing concentration, which could by caused by the increased intensity of intermolecular interactions forcing humic and fulvic particles or aggregates to contract into coils. However, it is necessary to analyze particle size distributions in detail, to compare intensity, volume, and number-based distributions, and to determine amounts of different size fractions in order to postulate any conclusions.

Considering all types of particle size distributions in Figure 3 (intensity, volume, and number-based), we can see that, in our study, humic acids contained relatively high numbers of small particles with a diameter <100 nm, while PPFA in alkaline solutions contained many particles with a diameter around 100 nm, and that particles of PPFA in water had two main fractions with average diameters of between 100 and 1,000 nm. However the volume occupied by particles bigger than 1 μm was much larger in comparison with the total volume occupied by smaller particles. On the basis of the obtained results, we decided to use volume based records in order to analyze particle size distributions in detail. A similar approach used by Esfahani et al. (2015) detected three populations in SRHA, SRFA and Aldrich humic acids (10–100, 100–1,000 nm, and >1 μm) on the basis of volume based distributions. Volume distributions were also preferred by Tarasevich et al. (2016) who analyzed the sodium salt of Aldrich humic acids and divided it into three fractions called nanoparticles (30–150 nm), submicroparticles (200–700 nm), and microparticles (1.6–5.6 μm).

Monitored changes in the volume based distributions with changing concentration obtained for humic substances isolated from the same source (the example for NLHA and NLFA in Figure 4) provided some interesting results. It seems that alkaline conditions supported the disaggregation and formation of smaller particles mainly in diluted solutions, while water as a neutral solvent supported the formation of big aggregates with a diameter ≥1 μm and led to the volumes of smaller particles being very low excepting highly concentrated fulvic solutions. Only nanoparticles and submicroparticles were detected for NLFA, SRFA and ESFA samples in concentrated aqueous systems. If the submicroparticles of fulvic acids were observed, their diameter was usually bigger both in NaOH and water than that of humic acids. The size of macroparticles was comparable (e.g., in the case of SRHA and SRFA) or bigger for fulvic acids (in most cases). Two mono-modal distributions were detected: the mostly diluted alkaline solution of NLFA contained only nanoparticles, and the aqueous solution of ESFA contained only macroparticles.

In general, three volume based particle populations were detected in humic and fulvic solutions. Average diameters of the particle populations are listed in Table 1.

Two main processes can affect the properties and behavior of humic and fulvic acids in solutions: the dissociation of acidic functional groups and the breaking up of humic aggregates into smaller molecular associations and/or molecules (Klučákov and Kalina, 2015; Klučáková and Věžníková, 2017). Important parameters affecting the spatial arrangement of the obtained humic and fulvic fractions were their concentration and pH. Fulvic particles in water were bigger than in alkaline solution as a result of suppressed dissociation. The decrease in particle sizes with increasing concentration was probably caused by the increased intensity of intermolecular interactions forcing humic and fulvic particles or aggregates to contract into coil. The bigger diameters of submicroparticles observed for fulvic acids in comparison with humic ones could be caused by higher amounts of ionizable functional groups allowing the greater expansion of fulvic coils in alkaline solution.

The measurement of zeta potential of humic and fulvic acids showed that the colloidal stability was higher for humic acids. Fulvic acids were unstable in water probably due to lower dissociation degree of their functional groups.

## Conclusions

In this study, several populations of humic and fulvic acids were investigated. Their number fluctuated from one to three depending on the used solvent (pH) and concentration. Small nanoparticles were detected in all alkaline solutions of humic and fulvic acids except for the most concentrated ESFA. In contrast, fulvic acids in water only contained small particles in concentrated systems. Large macroparticles or aggregates were observed in the majority of the studied systems. Exceptions with respect to the finding described in the previous sentence were recorded for some diluted humic solutions in NaOH and the most concentrated fulvic solutions in water. Medium fractions of fulvic submicroparticles had higher average diameters than those in humic acids. Colloidal stability increased generally with increasing concentration and pH.

## Author contributions

MK was involved in aspects of data acquisition, analysis, and interpretation. MK prepared the manuscript, approved the final version, and are accountable for the data presented and interpretation therein.

### Conflict of interest statement

The author declares that the research was conducted in the absence of any commercial or financial relationships that could be construed as a potential conflict of interest.
